# The Role of Epigenetic Modifications in Abdominal Aortic Aneurysm Pathogenesis

**DOI:** 10.3390/biom12020172

**Published:** 2022-01-21

**Authors:** Kevin Mangum, Katherine Gallagher, Frank M. Davis

**Affiliations:** Section of Vascular Surgery, Department of Surgery, University of Michigan, 5364 Cardiovascular Center, 1500 E. Medical Center Drive, Ann Arbor, MI 48109, USA; kevmangu@med.umich.edu (K.M.); kgallag@med.umich.edu (K.G.)

**Keywords:** macrophage, monocyte, inflammation, epigenetics, cardiometabolic, vascular

## Abstract

Abdominal aortic aneurysm (AAA) is a life-threatening disease associated with high morbidity and mortality in the setting of acute rupture. Recently, advances in surgical and endovascular repair of AAA have been achieved; however, pharmaceutical therapies to prevent AAA expansion and rupture remain lacking. This highlights an ongoing need to improve the understanding the pathological mechanisms that initiate formation, maintain growth, and promote rupture of AAA. Over the past decade, epigenetic modifications, such as DNA methylation, posttranslational histone modifications, and non-coding RNA, have emerged as important regulators of cellular function. Accumulating studies reveal the importance of epigenetic enzymes in the dynamic regulation of key signaling pathways that alter cellular phenotypes and have emerged as major intracellular players in a wide range of biological processes. In this review, we discuss the roles and implications of epigenetic modifications in AAA animal models and their relevance to human AAA pathology.

## 1. Introduction

Aortic aneurysms are a relatively common disease characterized by permanent dilation of the aorta with regional specificity [1]. Abdominal aortic aneurysms (AAA) are the most common form of this disease, with dilation typically presenting in the infrarenal region. Population ultrasound screening studies have determined that the prevalence of AAAs is 4–7% in males over the age of 65 and 1–2% in females, with some studies indicating AAA incidence is declining [2,3]. Aortic dilation is largely asymptomatic, but increased size is associated with increased risk of rupture and carries an excessively high mortality, with studies detailing approximately 200,000 deaths worldwide per year attributed to AAA rupture [4]. Current therapeutic options for AAAs are limited and include temporary monitoring of the aortic dimensions in order perform open or endovascular surgical repair when the diameter has attained sufficient expansion (greater than 5.5 cm in males or greater than 5.0 cm in females), which portends a high likelihood of rupture. Indeed, there is a lack of proven medical therapies that either prevent AAA development or limit aneurysm growth once diagnosed. The lack of effective therapies is associated with an inadequate understanding of the mechanisms that form AAAs.

Pathological risk factors for the development of AAAs include both modifiable and nonmodifiable risk factors for cardiovascular disease. The major epidemiological risk factors related to AAA include male gender, age of more than 65 years old, and a history of smoking [5,6]. Additionally, there is clear evidence that genetic factors are important in AAA development [7]. A previous case-control study reported that first-degree relatives of those with an AAA are at approximately 10-fold increased risk of developing an AAA compared to individuals without family history, after adjustment for age and sex [8]. Furthermore, population-based twin studies have estimated the heritability of AAA to be between 70 and 77% [9,10]. Recently, several studies have investigated the association of specific genetic markers with AAAs using either candidate gene association or genome-wide association analyses [7,11,12]. Additionally, a number of epigenetic factors also affect aneurysm formation [13]. Epigenetic mechanisms cause dysregulated expression of aneurysm genes without significant sequence variation, as is the case for genetic causes of disease. Epigenetic modes of regulation include DNA methylation, post-translational histone modifications, and non-coding RNA. Over the past decade, substantial work has been conducted on the mechanisms of epigenetic regulation of cellular activation in cardiovascular disease. Through characterization of human tissue samples and animal models, a complex picture of cellular processes has been shown to play pivotal roles in AAA development. In this brief review, we will discuss recent findings that provide mechanistic insight into the role of epigenetics in pathological development of AAAs. Additionally, we will highlight preclinical studies that seek to uncover novel therapeutic strategies for the treatment of these conditions.

## 2. Epigenetic Modifications

Epigenetics refers to developmentally or environmentally induced modifications that do not alter the genetic code but instead control how information encoded in DNA is expressed in a tissue- and context-specific manner. Epigenetic marks have traditionally been considered to be stable, potentially transmissible to progeny, and to underlie stable differentiation into various cell types that express markedly different patterns of gene expression. Recently, it has become clear that epigenetic chromatin marks are dynamically regulated in response to environmental cues. This has resulted in a shift in the usage of epigenetics to include transient changes in chromatin in response to external stimuli that control gene expression [14]. Overall, epigenetic changes can be classified into three main categories: (i) DNA methylation; (ii) post-translational histone modifications; (iii) non-coding RNA. Regarding DNA methylation, it is predominantly associated with transcriptional repression and is characterized by transfer of a methyl group to the cytosine ring of DNA by DNA methyltransferases (DNMTs) to form 5-methyl cytosine (5mC) [15]. DNMT3A and DNMT3B deposit de novo methylation marks, while DNMT1 is responsible for maintaining these marks since these marks must be re-established with each cell division [16]. In mammals, the vast majority of DNA methylation in somatic cells occurs at clusters of CpG dinucleotides termed CpG islands, and approximately 40% of genes contain these islands in their promoters [17].

In eukaryotes, DNA is packaged into repeating units called nucleosomes by wrapping around multimeric histone proteins. Each histone (H) protein is an octamer comprised of two sets of H2A, H2B, H3, and H4 proteins, with a single histone H1 linker protein between nucleosomes. Each histone subunit has an N-terminal “tail” containing a lysine (K) residue that protrudes away from the surface of the histone octamer, creating an exposed surface. Here, histone-modifying enzymes can regulate gene expression through the addition or removal of acetyl or methyl groups [18]. First, acetylation and deacetylation is facilitated by histone acetyltransferases (HATs) and histone deacetylases (HDACs), respectively. Histone acetylation is linked to transcriptional activity, whereas histone deacetylation is associated with transcriptional repression [19]. Similarly, methylation and demethylation of histones is achieved by histone methyltransferases (HMTs) and histone demethylases (HDMs), respectively [18]. Histone methylation can induce both transcriptional activation and repression, depending on the number and location of the methyl groups. An active transcriptional state is characterized by positive marks, such as di- or trimethylation (me2/me3) at H3K4, H3K36, and H3K79. A repressed transcriptional state manifests itself by increased markings at H3K9me2/me3 and H3K27me3 [20].

Lastly, non-coding RNAs—including miRNAs and long non-coding RNAs (lncRNAs)—do not directly affect chromatin architecture but play an essential role in post-transcriptional regulation of gene expression. lncRNAs are a large group of non-protein coding transcripts that are more than 200 nucleotides in length. Although the mechanisms by which lncRNAs regulate gene expression are still incomplete, emerging evidence has suggested that lncRNAs can control gene expression at multiple levels, including epigenetic control, transcription, RNA processing, and translation. Many lncRNAs have been found to be significantly enriched in the chromatin fraction, and a common function of these lncRNAs is to recruit chromatin-modifying complexes, including the Polycomb group (PcG) or Trithorax group (TrxG), to create a repressive chromatin state or an active chromatin state, as well as affect gene expression either in cis or in trans to distant target genes [21]. Alternatively, some lncRNAs can form RNA-protein complexes with transcription factors and influence the localization and activity of the transcription factors that they bind, subsequently regulating gene expression [22,23]. miRNAs are evolutionary conserved, small, non-coding single-stranded RNAs produced by multistep processes of transcription, nuclear export, and cytoplasmic cleavage [24]. miRNAs are endogenous RNAs of ~22 nt that post-transcriptionally repress the expression of target genes, usually by binding to the 3′ untranslated region of messenger RNA. The stepwise progression of miRNA maturation has already been established Lee et al. and Maegdefessel et al. [25,26]. It is estimated that >60% of all protein-coding genes are directly regulated by miRNAs [27]. Furthermore, a given miRNA may bind to and regulate more than one target, sometimes as a part of the same signaling pathway, adding multiple levels of regulation.

## 3. Epigenetic Modifications in Inflammatory Cells in AAAs

Histological analyses of AAA samples have revealed leukocytic infiltration, degradation of the extracellular matrix, and depletion of vascular smooth muscle cells (VSMCs) as three pathological hallmarks of AAAs. Although many different types of leukocytes have been detected in AAA tissues, macrophages are the most prominent cell type present in the aortic media and adventitia, with the majority of macrophages originating from circulating monocytes [28,29]. At this time, the contribution of tissue resident macrophages remains poorly defined. Recently, through the characterization of surgical samples and animal models, investigations have demonstrated a role of epigenetic modification in monocytes/macrophages within the pathogenesis of aortic aneurysm formation. Global methylation levels within peripheral blood mononuclear cells are significantly altered on CpG islands in AAA patients in comparison to controls and exhibit a positive correlation with increased aortic diameter (Table 1) [30]. Separately, investigations have also revealed fundamental roles of histone modification in AAA macrophage inflammation. Specifically, single-cell RNA sequencing of human AAA tissues identified increased expression of the histone demethylase JMJD3 in aortic monocyte/macrophages, resulting in upregulation of an inflammatory immune response. Mechanistically, interferon-β regulates JMJD3 expression via JAK/STAT, and JMJD3 induces NFkB-mediated inflammatory gene transcription in infiltrating aortic macrophages. Pharmacological or genetic inhibition of macrophage-specific JMJD3 was able to prevent AAA development and rupture [31]. Expression of HDACs, specifically class I and IIa, was found to be increased in comparison to control samples and colocalized with macrophages within the aortic wall. Using murine models, treatment with HDAC class I (MS-275) or class IIa (MC-1568) inhibitors reduced AAA incidence, decreased macrophage inflammation, and reduced proinflammatory mediators [32]. Lastly, miRNAs have also been shown to regulate macrophage function in murine models of AAAs. First, by directly targeting Chi311, miRNA-24 limits inflammation by inhibiting macrophage recruitment and survival, as well as blocking production of cytokines IL-8 and CCL2 by SMCs and macrophages [33]. Another important miRNA, miRNA 33, modulated the inflammatory response, as miRNA-33 deficiency resulted in decreased proinflammatory ‘M1’ gene expression, protease activity, and macrophage infiltration into the aortic wall [34]. MiRNA-33 knockout mice showed decreased AAA formation after either angiotensin II (AngII) or CaCl_2_ treatment [34].

Regrading other leukocytes, increasing evidence suggests that T-cell dysfunction, particularly decreased suppressive effects of CD4^+^ CD25^+^ T regulatory cells, drives AAA development [35]. Part of this change arises from epigenetic alterations, like DNA methylation, which is significantly lower in AAA patients than in healthy controls groups. Similarly, expression of DNMT1, DNMT3A, and methyl-CpG-binding domain 2 (MBD2), both involved in mediating DNA methylation and transcriptional repression, were also decreased in isolated T cells from patients with AAAs [36]. Separately, miR-155 was found to be significantly elevated in AAA samples compared to controls [37]. miRNA-155 has been shown to play an important role in promoting (chronic inflammation by enhancing T-cell development through the downmodulation of cytotoxic T-lymphocyte-associated protein) CTLA4. CTLA4 may suppress T-cells directly or indirectly via other cells, such as antigen-presenting cells, although both mechanisms are executed through the CD80 and CD86 coreceptors [38]. mRNA molecular interactions may represent an important functional axis controlling T-cell activity in AAA.

## 4. Epigenetic Modifications in Endothelial Cells in AAAs

### 4.1. Regulation of Endothelial Differentiation in AAAs by Chromatin Remodeling Enzymes

The role of endothelial cells (ECs) in AAA formation is not well described. However, there is emerging evidence that suggests that ECs, while not comprising the predominant cellular makeup of the aortic wall, also play an important role in disease pathogenesis [6] (Table 2). Within ECs, chromatin remodeling enzymes (CME) regulate gene expression by carrying out post-translational modifications, such as methylation and acetylation on key histones, thereby regulating the accessibility of transcription factors to promoter-enhancer regions. As such, CMEs are critical epigenetic regulators of gene expression, and several have been identified in AAA pathogenesis. BRG1, a CME that is part of the SWI/SNF chromatin remodeling complex, is an ATPase containing protein that mobilizes nucleosomes, thereby altering chromatin structure. Deletion of BRG1 is embryonically lethal, and multiple labs have shown that it is important in cardiovascular development [39]. In endothelial cells, BRG1 appears to promote AAA formation, as endothelial-specific deletion suppressed AAA formation and limited inflammation due to diminished macrophage recruitment [40]. Interestingly, other groups have shown that BRG1 facilitates endothelial-to-mesenchymal transition (EndMT) in cardiac fibrosis, as siRNA knockout of BRG1 in endothelial cells inhibited AngII-induced expression of fibrotic genes, such as COL1A1, COL1A2, and vimentin [41]. While these results were not in the context of AAA, there is evidence to indicate that fibrotic remodeling and upregulation of fibrotic genes characterizes advanced AAA [42,43]. It remains to be determined whether endothelial BRG1 positively regulates fibrotic changes observed in AAA formation and whether BRG1 promotes aneurysm formation.

HDAC9 was found to be another regulatory target of endothelial-to-mesenchymal transition. Specifically, HDAC9 knockout mice displayed a more endothelial-like phenotype characterized by increased expression of endothelial-specific gene expression, such as ICAM2 and decreased mesenchymal markers, such as SM22 [44]. In this study, HDAC9 promoted the endothelial-to-mesenchymal switch, and in vivo, endothelial-specific HDAC9 knockout mice had a decreased atherosclerotic lesion burden compared to control littermates. It is worthwhile to mention that this study assessed HDAC9 deletion in atherosclerosis, and whether it plays a role in endothelial cells in AAA remains to be studied.

### 4.2. Non-Coding RNAs That Regulate Endothelial Cell Function

In particular, endothelial-associated miRNAs have been identified in the context of AAAs. One such miRNA is miRNA-126. miRNA-126 was identified via bioinformatic analysis to target ADAM9, a family of transmembrane and secreted proteins that regulates cell trafficking, inflammation, and cancer [45]. ADAM9 expression was 33-fold higher in human aortic ECs treated with AngII versus the control. Interestingly, AngII repressed miRNA-126 expression, indirectly supporting the hypothesis that miR-126 may negatively regulate ADAM9, which was supported by siRNA knockdown miRNA-126 in ECs, which led to increased ADAM9 expression. In contrast, miR-126 overexpression repressed endothelial ADAM9 expression. In vivo, treatment of AngII-infused ApoE^−/−^ mice with miRNA-126 inhibited AAA formation. Further, in these same mice, ADAM9 expression was decreased in miR-126 mice compared to controls, an effect that may have been due to miRNA-126-mediated transcriptional silencing of ADAM9. In one study, miRNA expression profiles were compared in tissue samples from atherosclerotic AAA walls versus normal walls [46]. Interestingly, miR-126 was one of the miRNAs identified that were significantly upregulated in AAA tissue, as well as other endothelial-associated miRNAs, such as miRNA-20a, miRNA-27a, miRNA-92a, miRNA-221, and miRNA-222, members of the let-7 family, and miR-21. While their exact role in AAA formation has not been elucidated, there is evidence connecting some of these miRNAs to EC function. For example, miR-20a inhibits apoptosis of endothelial progenitor cells by targeting PTEN and ATG7 expression, while repressing endothelial migration [47,48]. miRNA-27a is expressed in ECs and promotes angiogenesis by negatively regulating SEMA6A, an anti-angiogenic protein [49]. Interestingly, in a rat model of pulmonary artery hypertension, miRNA-27a enhanced endothelial-to-mesenchymal transition [50]. However, the role of this miRNA in the context of AAA has not been studied and introduces the exciting work of studying miRNA regulation of endothelial-to-mesenchymal transition in AAA formation in general. miRNA-92a is secreted by endothelial cells and suppresses KLF4 expression in macrophages to promote atherosclerotic lesion formation [51]. Interestingly, in this study, miR-92a levels in plasma were negatively correlated with KFL4 expression in atherosclerotic lesions. However, miRNA-92a’s specific mechanism of action in AAA has not been examined. miR-221 is required for angiogenesis in zebrafish and regulates endothelial tip-cell proliferation and migration [52]. miRNA-221 promotes tip-cell behavior by negatively regulating expression of CDKN1B and PIK3R1. Interestingly, miRNA-222 was studied in a rat model of AAA formation, and its expression was increased in AAA tissues [53]. The effects of miRNA-222 on aneurysm pathogenesis were due to inhibition of EPC proliferation and migration through targeting of ADIPOR1 expression.

Limited data are available regarding endothelial-associated lncRNA that play a role in AAA pathogenesis. However, one well-known lncRNA, MALAT-1, appears to regulate downstream IL-6-mediated production of reactive oxygen species in endothelial cells. Overexpression of MALAT-1 in human aortic endothelial cells increased IL-6-induced expression of NOX2 via activation of the NOX2 promoter, leading to ROS production, which is a key feature in AAA formation [54]. Additional studies are necessary to evaluate whether MALAT-1 induces AAA in vivo.

While not directly proven to play a role in AAA, a number of other endothelial-associated lncRNAs have been identified that regulate EC function in other vascular diseases, such as atherosclerosis. Thus, it is worth mentioning some of these, which may also regulate AAA pathogenesis. GATA6-AS was identified in one study among lncRNAs upregulated during hypoxia. GATA6-AS silencing inhibited TGFβ2-mediated EndMT in vitro. In pulldown experiments with GATA6-AS, LOXL2 was identified as one binding protein. LOXL2 leads to H3K4me4 deamination, thereby resulting in transcriptional silencing via non-methylation. The authors found that about 70% of all genes deregulated by GATA6-AS showed increased expression after LOXL2 inhibition, implying a repressive effect of GATA6-AS on LOXL2, although subsequent experiments are required to confirm this. EndMT was inhibited by another lncRNA, H19, in a different study [55]. Briefly, H19 overexpression in vitro led to decreased expression of mesenchymal markers, such as SM22, while siRNA-mediated knockdown of H19 led to decreased expression of endothelial markers, such as VE-cadherin and CD31, and increased expression of SM22 and SMA. However, these findings were studied in diabetic retinopathy, a disorder characterized by pathological retinal angiogenesis.

Although initially identified in smooth muscle cells, SENCR has been shown to also regulate endothelial function [56]. SENCE overexpression led to endothelial cell differentiation and commitment in an in vitro model of EC cell differentiation, as well as upregulation of a subset of angiogenic-related genes. Further, SENCR expression was reduced in human peripheral artery disease and coronary artery disease vessel wall EC, implicating its role in these diseases. While the role of SENCR in AAA formation has not been examined directly, AAA patients were shown to have higher circulating levels of endothelial progenitor cells (EPCs) than non-AAA age-matched controls [57]. In another study, SENCR was found to stabilize vascular endothelial cell adheres junctions via its interaction with CKAP4, thereby leading to decreased cellular permeability [58].

## 5. Epigenetic Modifications in Smooth Muscle Cells and Fibroblasts in AAAs

### 5.1. Regulating Smooth Muscle Cell Differentiation

Epigenetic regulation of vascular smooth muscle cells (vSMC) has been shown to play key roles in a variety of vascular diseases, including hypertension, atherosclerosis, and restenosis after angioplasty. Recent evidence suggests that a similar process may also play a key role in vSMC in the development and progression of AAA (Table 3). Briefly, it is well-established that vSMC dedifferentiate into a proliferate and synthetic subtype that contributes to disease progression. During this process, mature, contractile SMC genes, including SM myosin heavy chain (SM-MHC), SM22, calponin, and SM alpha-actin (SMA), are “turned off”, while synthetic, proliferate genes, many of which are involved in regulating extracellular matrix protein turnover, are “turned on”. This process of dedifferentiation, also known as phenotypic modulation or switching, is largely due to epigenetic regulation of vSMC genes, and it is reasonable that such changes in SMC marker gene expression are induced during AAA formation. A variety of epigenetic factors in SMC may play a role in AAA pathogenesis and include DNA methylation, histone modifications, microRNAs, and long, non-coding RNAs.

Regarding histone modifications, a recent study comparing AAA and healthy aortic samples reported a wide variety of histone acetylation transferases that were significantly higher in disease [59]. KAT2B, KAT3A, KAT3B, and KAT6B were among the highest expressed in AAA tissue. KAT6A expression was shown to correlate with the contractile vSMC marker myosin heavy chain 11 (MYH11), as well as inflammatory CD3+ and vascular cell adhesion molecule 1 (VCAM-1)+ ECs.

Separately, miRNAs negatively regulate gene expression through mRNA degradation or translational inhibition. miRNA regulation of gene expression is highly complex because a single miRNA often regulates multiple genes that themselves participate in autoregulatory gene expression feedback loops. Recently, microarray profiling has identified several miRNAs enriched in vSMC that may play a role in AAA formation. One of the most widely studied miRNAs in vSMC is the miRNA-143/145 cluster, which has been implicated in vascular SMC biology and disease. Although studies are lacking regarding the effect of this miRNA cluster on AAA development, this miRNA cluster has been shown to inhibit SMC phenotypic modulation, thereby stabilizing the mature SMC phenotype [60]. Specifically, in different models of vascular injury in vivo models, miRNA-143/145 were decreased in injured arteries, and miRNA-143/145 appears to protect against restenosis after injury in a variety of models [61]. Thus, based on these collective studies, it is reasonable to hypothesize that miRNA-143/145 may inhibit AAA development and/or progression by preventing SMC dedifferentiation. Similarly, miRNA-126-5p promotes human aortic vSMC differentiation by directly negatively regulating VEPH1 expression, thereby leading to increased expression of mature, contractile genes, such as SMA and MYH11, and decreased expression of synthetic genes, like vimentin and PCNA [62]. Furthermore, overexpression of miRNA-126-5p prevented Ang-II upregulation of MMP-9 and MMP-2, two MMPs responsible for matrix degradation and aneurysm formation. In a prior study, these same authors found that VEPH1 induced SMC dedifferentiation, providing overall support of the miRNA-126-5p/VEPH1 regulatory axis in regulating SMC phenotypic modulation in AAA formation [62]. In another study, miRNA-23b overexpression in ApoE^−/−^ mice infused with Ang II prevented AAA formation in this model [63]. Further, maintenance of the mature vSMC contractile phenotype was promoted by miRNA-23b, which targets and negatively regulates FoxO4 [64]. Further, in separate studies, miRNA-23b was shown to inhibit phenotypic modulation and neointimal hyperplasia in a carotid model of restenosis post balloon angioplasty [65].

### 5.2. miRNAs Regulating SMC Matrix Components

Other miRNAs have been shown to regulate the expression of key components involved in the synthesis and degradation of the vessel-wall matrix. One study examined the role of miR-516a-5p and the MTHFR/homocysteine pathway, which has been implicated in AAA pathogenesis. This study showed that miRNA-516a-5p overexpression inhibited MTHFR expression, while knockdown increased MTHFR expression. miR-516a overexpression also led to increased expression of MMP-2 and decreased expression of TIMP-1, two regulators of matrix pathway components in vSMC [66]. Notably, miRNA-516a was also upregulated in AAA explant tissue compared to healthy controls, as measured by microarray and validated by qPCR [67]. Another group profiled miRNA expression in the Angiotensin II (AngII) ApoE^−/−^ mouse model of AAA and found that miRNA-126a-5p was downregulated eight-fold in AAA tissue and decreased AAA formation in mice when overexpressed. Interestingly, overexpression of this miRNA inhibits ADAMTS-4, a proteinase that leads to matrix degradation [68].

### 5.3. miRNAs Regulating SMC Apoptosis and Proliferation

miRNAs have also been shown to regulate SMC apoptosis, which may represent another mechanism underlying AAA formation. miRNA-504 inhibits SMC apoptosis and may contribute to AAA formation [69]. This study showed that miRNA-504, which is upregulated in healthy aortic tissue, is downregulated in AAA tissue and limits p53-induced SMC apoptosis. Another group found that one miRNA, miRNA-15b-5p, led to vascular SMC apoptosis by negatively regulating expression of ACSS2, which happens to be downregulated in AAA tissue [70]. Another miRNA, miRNA-96-5p, induced SMC apoptosis and inhibited proliferation and migration by targeting and negatively regulating NFAT5 [71]. In similar studies, vSMC senescence, which is increased in AAA, was induced by miRNA-199a-5p and further stimulated by AngII. The authors found that negative regulation of Sirt1 expression by miRNA-199a resulted in increased ROS production and cellular senescence [72].

### 5.4. Long, Non-Coding RNAs Regulating SMC Function and Phenotypic Modulation

Several long, non-coding RNAs (lncRNAs) that regulate SMC function and differentiation have been identified in AAA pathogenesis. One lncRNA, PVT1, is upregulated in AAA tissue and appears to promote AAA formation by driving AngII-induced SMC phenotypic modulation and expression of MMPs in vitro, as well as in the AngII ApoE^−/−^ murine AAA model [73]. Interestingly, these effects were reversed by knockdown of PVT1 in vitro and in vivo, identifying PVT1 as a potential target for treatment of AAA. In a different study, RNA transcripts from murine AAA were profiled to identify non-coding RNAs underlying aneurysm formation. One of the most highly expressed lncRNAs, H19, increased SMC apoptosis when overexpressed. Downstream target analysis revealed HIF1α as a potential target of H19, and in vitro experiments revealed that this H19 increased HIF1α transcription via recruitment of the transcription factor Sp1 to the HIF1α promoter [74]. Another group screened several lncRNAs that were known to be differentially expressed in AAA and found that CRNDE was downregulated in AAA [75,76]. Overexpression of CRNDE in cultured vSMC increased proliferation and inhibited apoptosis. The authors found that CRNDE inhibited Smad3 ubiquitination and subsequent degradation, thereby leading to increased vSMC proliferation. Further, lentiviral overexpression of CRNDE in AngII-treated ApoE^−/−^ mice inhibited AAA growth. Related to this, the lncRNA SENCR was found to be downregulated from AAA tissues from AngII-treated ApoE^−/−^ mice [77]. siRNA knockdown of SENCER in cultured vSMC increased SMC apoptosis and expression of MMP-2 and MMP-9. In contrast, overexpression of SENCR decreased AAA formation in mice, suggesting that this lncRNA plays a protective role in the prevention of aneurysm formation. While other lncRNAs have hypothesized roles in AAA development, the majority of these studies are limited to in vitro experiments in SMC, and in vivo data are lacking. Future experiments with validated animal models will confirm the role of these lncRNAs in AAA pathogenesis.

Epigenetic modifications, such as DNA methylation and post-translational modification of histones, play significant roles in regulation of gene expression, and a variety of emerging studies have shown that these influence AAA formation and progression [13,78]. Generally, DNA methylation within gene promoters is associated with transcriptional repression. Toghill et al. assessed global DNA methylation in peripheral blood mononuclear cells in patients with AAA compared to patients without AAA and found the percentage of DNA methylation positively correlated with AAA diameter [30]. Approximately 80% of DNA methylation occurs at CpG islands within gene promoters. CpG islands are defined by a cytosine nucleotide, followed by a guanine nucleotide, and are key sites of transcriptional regulation. In this study, several “hit” genes showed differential CpG methylation. Of these genes, SMYD2 was differentially expressed in vSMCs isolated from AAA versus control tissue, and DNA methylation within the SMYD2 promoter was negatively correlated with SMYD2 expression. Additional genes that showed differential DNA methylation included CNN2 and SERPINB9, and both of these showed increased expression in aneurysm tissue that was enriched in the SMC layer via immunohistochemical tissue staining [35].

## 6. Conclusions

The past two decades have shown tremendous growth in the understanding of molecular mechanisms underlying AAA pathobiology. Evidence discussed herein suggests that epigenetic processing plays a central role in the pathogenesis of AAA development. Indeed, DNA methylation, post-translational histone modifications, and non-coding RNAs have all been shown to regulate structural and inflammatory cells that contribute to pathological aortic dilation. Future investigations should be geared toward improved understanding of cell-type-specific mechanisms of disease since a significant number of reports analyze whole aortic tissue rather than specific cell types from aneurysm samples. Further, pharmacological intervention to reprogram a ‘diseased’ epigenetic landscape is an attractive therapeutic target for the treatment of cardiovascular disorders. The understanding of chromatin architecture has led to the design of specific molecules to modulate chromatin accessibility by enhancing or repressing epigenetic marks on DNA/histone complexes [79]. The challenge for future investigations concerns how to achieve tissue-specific modulation of chromatin with an efficient and specific delivery system since systemic inhibition or activation may lead to adverse side effects. With future research, epigenetics will serve as a useful framework through which to gain new insights into the pathogenesis of AAAs.

## Figures and Tables

**Table 1:** Epigenetic modifications in inflammatory cells in AAAs.

Epigenetic Modification	Cellular Origin	Regulation	Target Gene(s)	Related Function
*DNA Methylation*				
DNMT1, DNMT3A	T-lymphocyte	Downregulated	---	Potential role in T-cell dysfunction, particularly decreased suppressive effects of CD4 + CD25+ T regulatory cells
*Histone Modification*				
JMJD3	Macrophage	Upregulated	H3K27me3 on promoters for: IL1β, TNF, IL23	Increases macrophage inflammatory cytokine production; macrophage proinflammatory polarization
HDAC I and IIa	Macrophage	Upregulated	---	Increased proinflammatory macrophage phenotype and inflammatory mediators
*Non-coding RNA*				
miRNA-24	Macrophage	Upregulated	Chi311	Limits inflammation and ECM degeneration; overexpression reduces AAA
miRNA-33	Macrophage	Downregulated	ABCA1	Monocyte chemotaxis, macrophage accumulation; inhibition reduces AAA
miRNA-181b	Macrophage	Upregulated	TIMP3, ELN	Downregulates ECM
miRNA-223	Macrophage	Upregulated in tissue, downregulated plasma	MMP12	Inhibits vascular inflammation
miRNA-155	T-lymphocyte	Upregulated	CTLA4	Regulation of T-cell activation

AAA, abdominal aortic aneurysm; ECM, extracellular matrix; IL1β, interleukin 1β; IL23, interleukin 23; TNF, tumor necrosis factor.

**Table 2:** Epigenetic modifications in endothelial cells in AAAs.

Epigenetic Modification	Cellular Origin	Regulation	Target Gene(s)	Related Function
*Histone Modification*				
BRG1	Endothelial cell		COL1A1, COL1A2, vimentin	Promotes AAA formation upregulation of fibrotic gene expression
HDAC9	Endothelial cell			Enhances endothelial-to-mesenchymal transition
*Non-coding RNAs*				
miRNA-20a	Endothelial cell	Upregulated	PTEN, ATG7	Inhibits endothelial cell apoptosis
miRNA-21	Endothelial cell	Upregulated		
miRNA-27a	Endothelial cell	Upregulated	SEMA6A	Promotes endothelial-to-mesenchymal transition
miRNA-92a	Endothelial cell	Upregulated	KLF4	Secreted by EC, inhibits KLF4 expression in macrophages; enhances atherosclerotic lesion formation
miRNA-126	Endothelial cell	Upregulated	ADAM9	Overexpression reduces AAA formation; suppression of inflammatory cytokines
miRNA-221	Endothelial cell	Upregulated	CDKN1B, PIK3R1	Promotes angiogenesis via regulating endothelial tip-cell proliferation and migration
miRNA-222	Endothelial cell	Upregulated	ADIPOR1	Overexpression promotes AAA by interfering with endothelial progenitor cell function
Let-7 (miRNA)	Endothelial cell	Upregulated		
GATA6-AS (lncRNA)	Endothelial cell	Upregulated?	LOXL2	Inhibition of TGFβ2-mediated endothelial-to-mesenchymal transition
H19 (lncRNA)	Endothelial cell			Inhibition of endothelial-to-mesenchymal transition
MALAT-1 (lncRNA)	Endothelial cell		NOX2	Increased inflammation and ROS production
SENCR (lncRNA)	Endothelial cell		CKAP4	Stabilizes vascular EC adherens junctions

AAA, abdominal aortic aneurysm; EC, endothelial cell; ROS, reactive oxygen species.

**Table 3:** Epigenetic modifications in smooth muscle cells in AAAs.

Epigenetic Modification	Cellular Origin	Regulation	Target Gene(s)	Related Function
*Non-Coding RNAs*				
miRNA-15b-5p	SMC	Upregulated	ACSS2	Promotes SMC apoptosis
miRNA-23b	SMC		FOXO4	Inhibits AAA formation; maintains SMC mature phenotype, inhibits phenotypic modulation
miRNA-96-5p	SMC		NFAT5	Promotes SMC apoptosis
miRNA-126-5p	SMC	Downregulated	VEPH1	Inhibits AAA formation; overexpression inhibits MMP-9 and MMP-2 expression and promotes SMC differentiation
miRNA-143/145	SMC	Downregulated	SRF, myocardin, KLF4/5, MRTF-B	Inhibits phenotypic modulation in SMC after vessel injury
miRNA-199a-5p	SMC	Upregulated	SIRT1	Increases SMC senescence and ROS production
miRNA-504	SMC	Downregulated	p53	Inhibits p53-induced SMC apoptosis
miRNA-516a	SMC	Upregulated	MTHFR	Overexpression leads to increased expression of MMP-2 and decreased expression of TIMP-1
CRNDE	SMC	Downregulated	SMAD3	Increases SMC proliferation, inhibits SMC apoptosis; inhibits AAA progression
H19	SMC	Upregulated	HIF1α	Increases SMC apoptosis
PVT1	SMC	Upregulated		Induces SMC phenotypic modulation
SENCE	SMC	Downregulated		Increases SMC apoptosis and expression of MMP-2 and MMP-9; decreases AAA formation
SMYD2	SMC	Downregulated		Increased promoter methylation in AAA

AAA, abdominal aortic aneurysm; SMC, smooth muscle cell.
